# Changes in ionizing radiation dose rate affect cell cycle progression in adipose derived stem cells

**DOI:** 10.1371/journal.pone.0250160

**Published:** 2021-04-27

**Authors:** Matthew Rusin, Nardine Ghobrial, Endre Takacs, Jeffrey S. Willey, Delphine Dean

**Affiliations:** 1 Bioengineering Department, Clemson University, Clemson, South Carolina, United States of America; 2 Physics and Astronomy Department, Clemson University, Clemson, South Carolina, United States of America; 3 Department of Radiation Oncology, Wake Forest School of Medicine, Winston-Salem, North Carolina, United States of America; Chonnam National University Medical School, REPUBLIC OF KOREA

## Abstract

Biomedical use of radiation is utilized in effective diagnostic and treatment tools, yet can introduce risks to healthy tissues. High energy photons used for diagnostic purposes have high penetration depth and can discriminate multiple tissues based on attenuation properties of different materials. Likewise, the ability to deposit energy at various targets within tumors make the use of photons effective treatment for cancer. Radiation focused on a tumor will deposit energy when it interacts with a biological structure (e.g. DNA), which will result in cell kill should repair capacity of the tissue be overwhelmed. Likewise, damage to normal, non-cancerous tissues is a consequence of radiation that can lead to acute or late, chronic toxicity profiles. Adipose derived stem cells (ADSCs) are mesenchymal stem cells that have been proven to have similar characteristics to bone marrow derived stem cells, except that they are much easier to obtain. Within the body, ADSCs act as immunomodulators and assist with the maintenance and repair of tissues. They have been shown to have excellent differentiation capability, making them an extremely viable option for stem cell therapies and regenerative medicine applications. Due to the tissue ADSCs are derived from, they are highly likely to be affected by radiation therapy, especially when treating tumors localized to structures with relatively high ADSC content (eg., breast cancer). For this reason, the purpose behind this research is to better understand how ADSCs are affected by doses of radiation comparable to a single fraction of radiation therapy. We also measured the response of ADSCs to exposure at different dose rates to determine if there is a significant difference in the response of ADSCs to radiation therapy relevant doses of ionizing radiation. Our findings indicate that ADSCs exposed to Cesium (Cs 137)-gamma rays at a moderate dose of 2Gy and either a low dose rate (1.40Gy/min) or a high dose rate (7.31Gy/min) slow proliferation rate, and with cell cycle arrest in some populations. These responses ADSCs were not as marked as previously measured in other stem cell types. In addition, our results indicate that differences in dose rate in the Gy/min range typically utilized in small animal or cell irradiation platforms have a minimal effect on the function of ADSCs. The potential ADSCs have in the space of regenerative medicine makes them an ideal candidate for study with ionizing radiation, as they are one of the main cell types to promote tissue healing.

## Introduction

Exposure to ionizing radiation is unavoidable as there are plenty of natural background radiation sources and exposure through imaging sources is a standard part of modern medical practice. However, adverse effects from ionizing radiation can be limited by minimizing exposure. Additionally, dose rate of exposure is a potential contributor to cell, tissue, and organism response to radiation. The relationship between dose rate and adverse biological effects such as induction of cancer or normal tissue toxicity is complicated. For instance, dose rate has been directly linked to elevated lifetime risk of cancer and non-cancer disease formation [1,2]. These observations are, in part, based off the Lifespan Study (LSS) of atomic bomb survivors who were exposed to various total doses of radiation (depending on the distance from the bomb) at a very high dose rate. The dose rate for background radiation is approximately 2-4mSv/yr depending upon location, which generally would be considered a dose rate that will result minimal toxicity [3,4]. Diagnostic medical imaging machines, such as computed tomography (CT), use much higher doses of radiation. Depending on the location of the scan, a patient can receive between 2-20mSv over a period of several minutes [5,6], which is a considerably greater rate than observed from background radiation and has been shown to be associated with elevated cancer risk [7,8].

Radiation exposure is commonly used as a treatment for various forms of cancer. For conventional radiation therapy procedures, a patient can receive between 40-60Gy given in daily, fractionated doses of ~1.8Gy [9]. Fractionation is generally employed in conventional radiation therapy to limit dose to normal tissues; however, the total absorbed dose and dose rate delivered per fraction during these therapies is many times higher than from medical diagnostic imaging, which can and does increase the risk for secondary cancers or normal tissue toxicity [2,6,10]. The effects of dose rate used during conventional radiation therapy and radiosurgical procedures (e.g., gamma knife) on biologic outcomes and toxicities is controversial. For preclinical studies, e.g. rodent models for treatment of disease using orthovoltage X-rays or cell based work using small animal-radiation platforms or a radioactive isotope source (e.g. cobalt-60 or cesium-137), the dose rate is generally not considered as being consequential for radiobiologic purposes.

Numerous *in vitro* studies have been performed that show most cell types exhibit hallmarks of damage detection and repair (activation of the p53 pathway, cell cycle arrest, apoptosis, immune system activation, etc.) up to 2 days following radiation exposure [11–14]. *Rødningen et al* found correlation between activated genes and the number of doses a sample received [15]. Comparison experiments on the effects of various dose rates on adipose derived stem cells (ADSC) are surprisingly sparse, with most comparing dose rates that are orders of magnitude different (e.g. mGy/min vs Gy/min) [16,17]. While it is important to understand the different responses cells have to very low vs high dose rate radiation, these experiments on large variations of dose rates use a wide range of different sources that have different uses. For instance, brachytherapy dose rate is varied depending on the sources used and can vary between 0.08Gy/h to 12Gy/h. [18] On the other hand, dose rate for gamma knife procedures is 2-3Gy/min [19].

This study aims to understand the differences in ADSCs response to two different dose rates from the same gamma irradiation source. The results can shed light on how ADSCs react to ionizing radiation exposure, and if different dose rates in the same clinically relevant range can elicit a different response.

## Materials and methods

### Cell culture and gamma irradiation

Primary human adipose-derived mesenchymal stem cells (hADSCs) were acquired from ATCC (ATCC PCS-500-011, Manassas, VA) and cultured in high glucose Dulbecco’s Modified Eagle Medium (Corning; Corning, NY) with 10% Fetal Bovine Serum (Atlanta Biologicals; Flowery Branch, GA) and 1% penicillin-streptomycin (HyClone; Logan, Utah). These hADSCs were selected as they are known to differentiate along several lineages and they are often used for regenerative medicine or tissue engineering applications in the literature. One day prior radiation exposure, cells were passaged and counted using a Scepter^™^ (EMD Millipore; Darmstadt, Germany) automated cell counter to ensure the proper cell density was plated for each experiment. Depending on the experiments being performed during that trip, cells were either plated in T25, 6- or 24-well plates.

During travel from the facilities at Clemson University to the radiation facility at Wake Forest School of Medicine (~4 hours), cell culture systems were kept in a portable incubator capable of maintaining proper temperature. Upon arrival, all cell culture systems were placed in a standard incubator (37°C, 5% CO_2_) for 1 hour to re-acclimate the cells and media. After this period, cells were irradiated with a custom Cs^137^ gamma irradiator for a total dose of 2Gy and either a lower dose rate (LDR; 1.40Gy/min) or a higher dose rate (HDR; 7.31Gy/min), then placed back in the portable incubator for travel back to Clemson University. Control group cells traveled to the radiation facility but received 0Gy of radiation. Note that the low dose rate for this study is much higher than those from some applications, such as brachytherapy. It is comparable to the low dose rates used in gamma knife.

### Proliferation assay

Proliferation changes were measured in hADSCs after radiation exposure using CellTiter96^®^ Aqueous One Solution (Promega; Madison, WI). This solution is an MTS based, colorimetric assay that measures the formazin product released by cells in culture. The formazin product is directly proportional to the number of live cells in culture, therefore it is possible to calculate the total cells by converting the absorbance value. ADSCs were plated at 2,500cells/cm^2^ in 24-well plates 1 day before radiation exposure to allow the cells time to adhere. After radiation exposure, the plates were returned to the incubator, and the assay was performed at 6 hours, 1 day, 3 days, and 5 days post exposure. The old media was first removed from the wells, then 0.5mL of fresh media and 100μL of CellTiter96^®^ was added. The plates were then incubated at 37°C and 5% CO_2_ for 1 hour, then had absorbance read at 490nm using a Synergy H1 Biotek plate reader.

### Flow cytometry

Cell cycle and apoptosis distribution were determined using a Guava easyCyte Flow Cytometer (EMD Millipore). Cellular DNA was stained using a Guava Cell Cycle Reagent (EMD Millipore) containing propidium iodide (PI) to visualize what phase of the cell cycle each cell is in. The fluorescent intensity of PI-stained DNA increases with each progressive phase, allowing a clear indication of the percentage of cells present in each phase.

hADSCs were passaged and then plated at 5000cells/cm^2^ in 6-well plates 1 day before radiation exposure. After exposure, cells were removed from the culture surface and collected at day 1, 2, and 3 using 0.25% Trypsin in EDTA (Corning). Cells were pelleted by centrifugation. After removing the supernatant, cells were resuspended and washed in sterile 1X Phosphate Buffered Saline (PBS; MP Biomedicals; Solon, OH) and counted. Cell suspension was then plated in a 96-well U-bottom plate at a density of 5.0x10^4^ cells per well. The plate was centrifuged and the supernatant carefully removed so as not to disturb the pellet. The pellet was broken up by trituration in residual PBS, then 200μl of 70% ice-cold ethanol was added dropwise to each well while gently swirling the plate to fix the cells. Fixed cells were washed with 1X PBS to remove ethanol residue, centrifuged and resuspended in 200μl of Guava Cell Cycle Reagent. After 30 minutes of incubation at room temperature, the plate was read using the flow cytometer.

The presence of apoptotic cells was determined using the Guava Mitochondrial Depolarization Assay kit (EMD Millipore), which uses a fluorescent cationic dye, JC-1. This dye collects in the mitochondria of healthy cells. When a cell becomes apoptotic, the membrane of the mitochondria will become depolarized, allowing JC-1 to leak into the cytoplasm where it’s broken down, changing the color it fluoresces. The kit also includes a solution of 7-AAD which enters cells and fluoresces when they die. 7-AAD cannot enter live cells because their membrane has not been compromised. hADSCs were plated at 2,500cells/cm^2^ 1 day before radiation exposure in 6-well plates. After exposure, cells were removed from culture surface using 0.25% Trypsin in EDTA, centrifuged and resuspended in 200μl media. This cell suspension was transferred to a 96-well flat bottom plate where it was mixed with 4μl of a JC-1/7-AAD solution. The plate was then incubated for 30 minutes at 37°C and read using the flow cytometer.

### Reverse Transcription Polymerase Chain Reaction (RT-PCR)

hADSCs were seeded at 5,000cells/cm^2^ in 6-well plates 1 day prior to radiation exposure. Total RNA was collected from irradiated cells at 4 hours, 1, 2, and 3 days post exposure using TRIzol Reagent (Invitrogen; Waltham, MA). RNA purification was carried out per the manufacturer’s instructions. Single-stranded cDNA was obtained using the High Capacity cDNA Reverse Transcription Kit (Applied Biosystems; Waltham, MA) following the manufacturer’s protocol. cDNA was then amplified in PCR using PowerUp SYBR Green Master Mix (Applied Biosystems) and specific primers (CD44 and TP53 from Integrated DNA Technologies, Coralville, IA). Amplification was performed using a StepOnePlus Real-Time PCR System (Applied Biosystems) with appropriate settings based upon the SYBR Green protocol.

### Immunocytochemistry

Cells for immunocytochemistry were cultured and irradiated in T25 culture flasks at 1,500cells/cm^2^. Following radiation exposure, cells were trypsinized and reseeded onto poly-lysine-coated Flourodishes (World Precision Instruments; Sarasota, FL) at 2,500cells/cm^2^. Fixation and staining was done 1, 2, and 3 days post irradiation. Fixation was done with 4% paraformaldehyde for 30 minutes at 37°C. Cells were permeablized using 0.3% Triton-X (Sigma-Aldrich; St. Louis, MO) for 15 minutes at room temperature, followed by blocking with 5% BSA for 1 hour. After rinsing with PBS, cells were incubated with 2μg/ml of either monoclonal mouse anti-p21/WAF1/Cip1 (Clone CP74; EMD Millipore) or monoclonal mouse anti-p53 (Clone PAB1801; Invitrogen) overnight at 4°C. Secondary blocking was done with 5% donkey serum for 30 minutes at room temperature. Primary antibodies were then tagged using 4μg/ml donkey anti-mouse Alexa Fluor 647 secondary antibody (Invitrogen) for 1 hour at room temperature in the dark. All samples were then counter stained using Alexa Fluor 488 phalloidin and DAPI according to manufacturer’s protocols. Imaging was performed using an Olympus IX81 spinning disk confocal microscope (Olympus; Tokyo, Japan), while collection and post-processing was done using MetaMorph Image Analysis software (Molecular Devices; Sunneyvale, CA).

Antibody expression intensity was analyzed using a custom MATLAB program that separated the image based upon the location of the nucleus, and then compared the intensity inside the nucleus with the cytoplasmic intensity.

### Senescence

hADSCs were also fixed and stained for β-galactosidase (β-gal) to check for cellular senescence. Cells seeded in 6-well plates at 2,000cells/cm^2^ were fixed 4 hours, 1, and 2 days after radiation exposure, then stained using a Senescence Detection Kit (BioVision; Milpitas, CA) following the manufacturer’s protocol. After staining, cells were imaged using an EVOS Cell Imaging System (Advanced Microscopy Group; Bothell, WA).

### Statistical analysis

All experimental conditions were performed with a minimum of 3 replicates. Data was collected and analyzed for statistical significance by a One-Way ANOVA (p<0.05) using JMP Pro 12 (SAS; Cary, NC).

## Results

### Changes in proliferation

**Fig 1:**
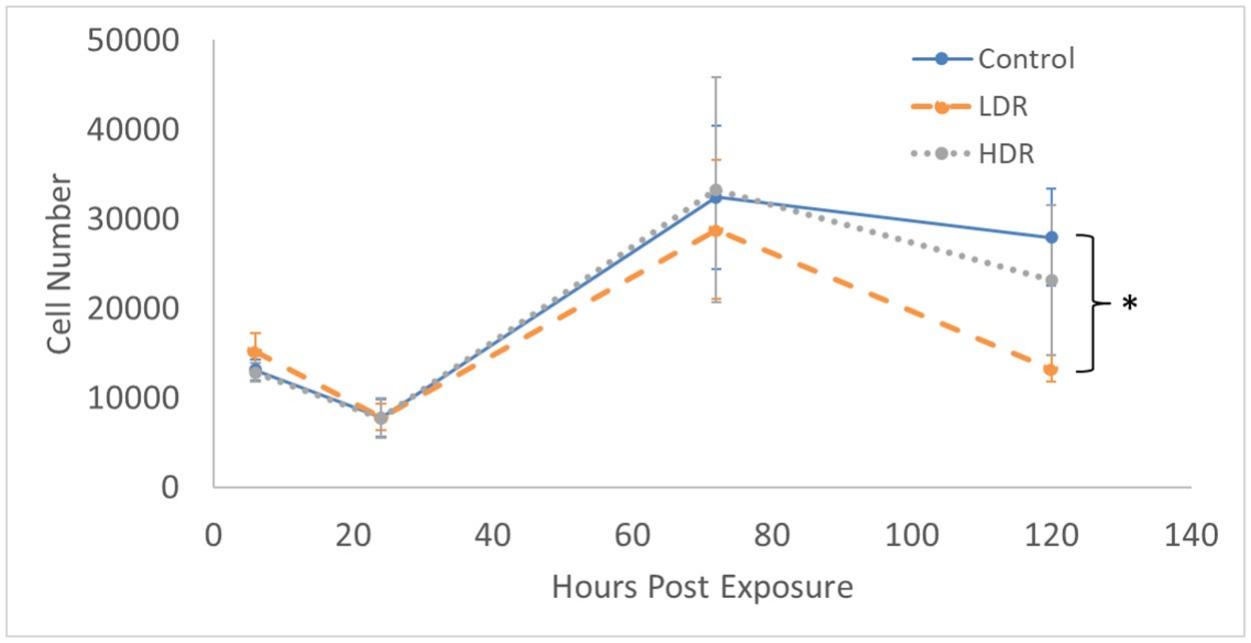
Total cell number present in culture after 2Gy gamma irradiation. N = 3 independent experiment for each time point and * indicates that the point is statistically different (p<0.05) from the control at the same day.

**Fig 2:**
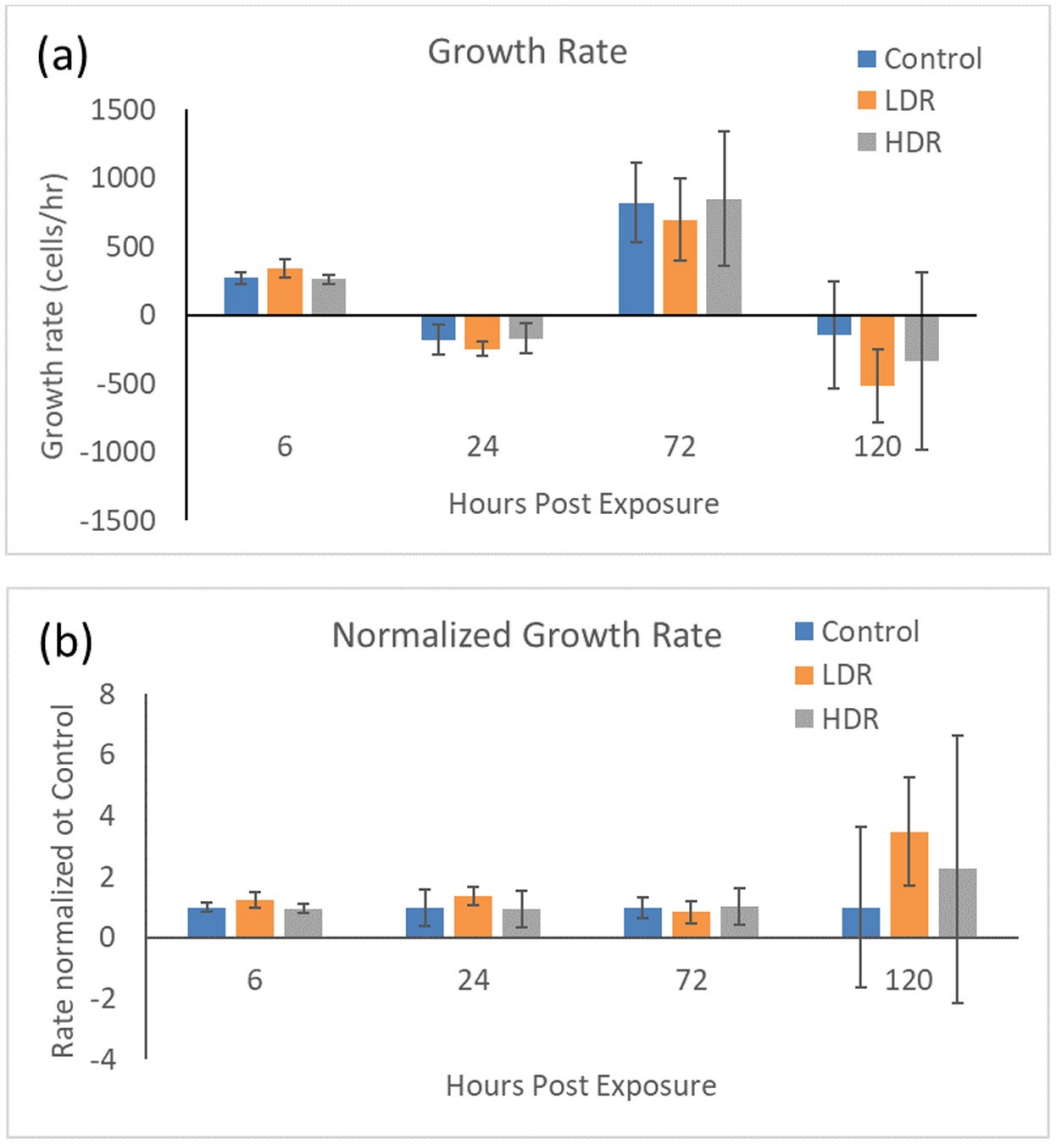
a) Cell growth rate after 2Gy gamma irradiation. b) Irradiated sample growth rates normalized to control at each time point. N = 3 independent experiments for each time point.

Exposure to 2Gy ionizing radiation at different rates had minimal effect on the proliferation of ADSCs. There is no statistical difference in the number of cells in culture expect for LDR at day 5 where it is significantly different from control, but not HDR irradiated samples (Fig 1). Similarly, the proliferation rate between samples shows no differences (Fig 2). The rate of proliferation (Fig 2a) was determined by taking the difference of total cell number between the current and previous time points and dividing by the time elapsed; Fig 2b shows the proliferation rate normalized to the proliferation rate of the control condition over time.

### Cell cycle distribution

**Fig 3:**
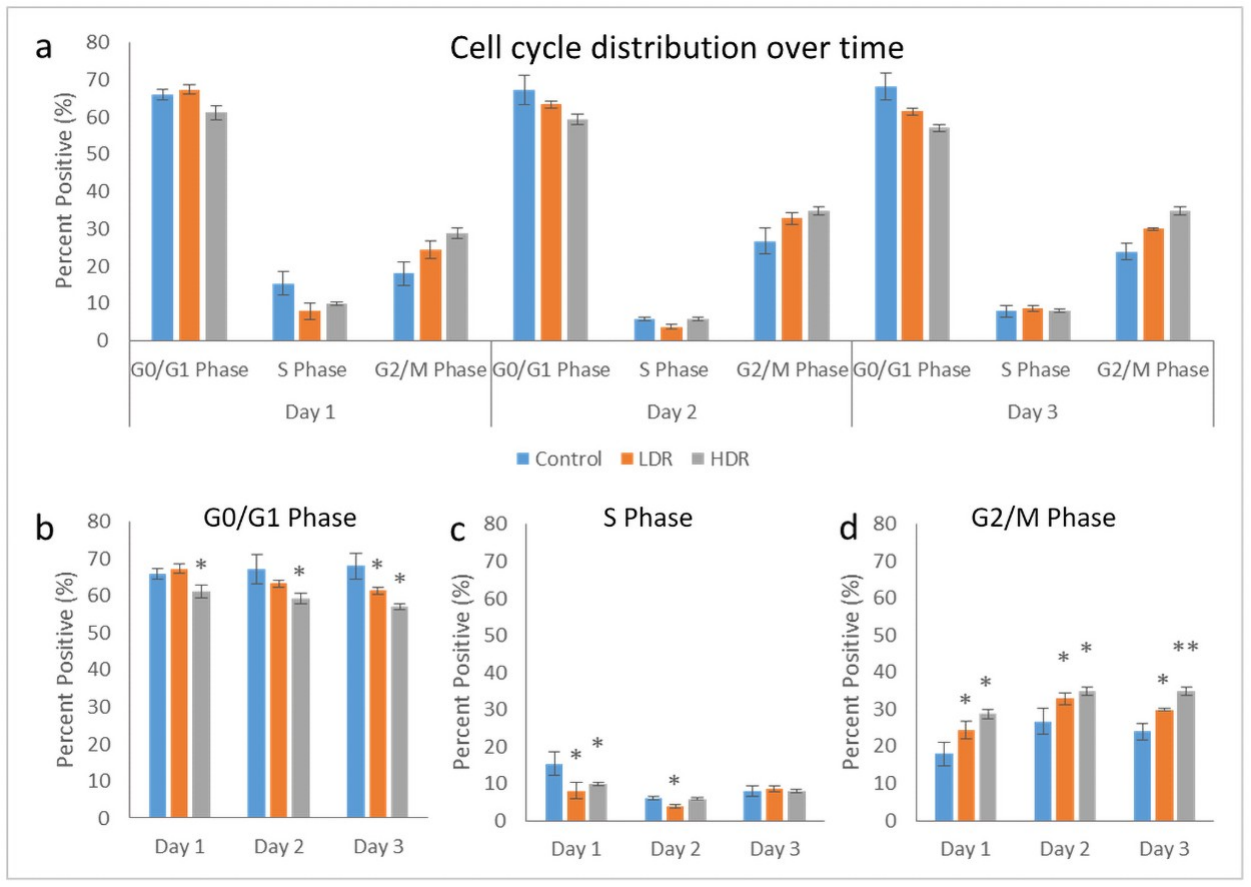
a) Cell cycle distribution after 2Gy gamma irradiation. Separated phase analysis, b) G0/G1 phase, c) S phase, d) G2/M phase. * = p<0.05 relative to the control from the same time point, N = 4 separate independent trials of each time point and condition.

Cell cycle analysis by flow cytometry was used to determine the percentage of cells present in each phase of the cell cycle: G_0_/G_1_, S, or G_2_/M. Using the Guava Cell Cycle Reagent, fixed 2Gy irradiated cells were stained and analyzed for differences in cell cycle distribution between LDR and HDR. Fig 3a shows the full distribution for all samples over the collected time points. At all the time points, HDR samples in G_0_/G_1_ were significantly different than both control and LDR samples, while LDR was significantly different than control at day 3 only (Fig 3b). LDR and HDR samples in S phase were significantly different from controls at day 1, and LDR was different from both control and HDR at day 2 (Fig 3c). There was no difference between samples in S phase at day 3. LDR and HDR samples in G_2_/M phase were significantly different from control at all time points, and LDR and HDR were different from each other at day 3 (Fig 3d). It should be noted that there were no differences in cell viability between the control cells that traveled to the radiation facility and those that stayed in the standard incubator environment.

### Apoptosis induction

**Fig 4:**
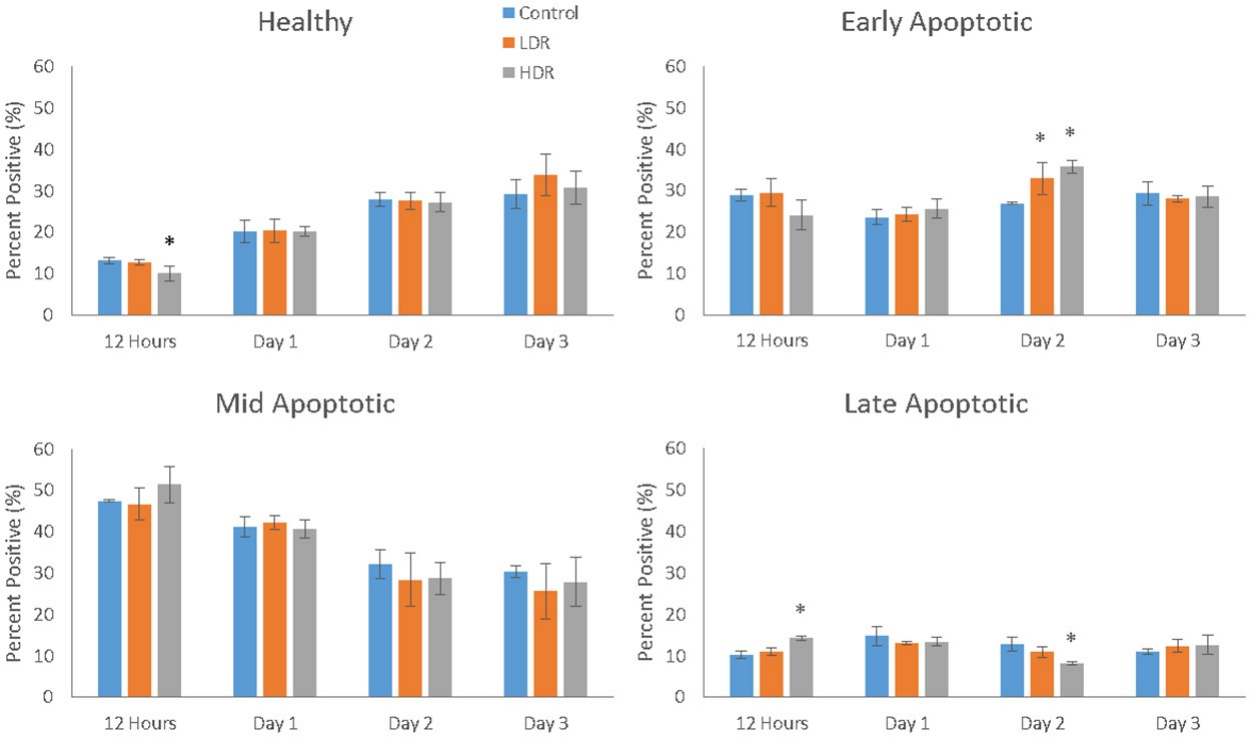
Cell distribution within the various stages of apoptosis as measure by mitochondrial membrane depolarization (* = p<0.05 when compared to the control for the same time point). N = 3 independent experiments each with 2000 cells that analyzed for each time point and condition.

Depolarization of the mitochondrial membrane was used as the determining factor for the stage of apoptosis a cell may be in (healthy, early-, mid-, or late-apoptotic). A more depolarized membrane potential corresponds to a later stage of apoptosis. As seen in Fig 4, the proportion of healthy cells grew over time, but HDR samples 12 hours after exposure were significantly different from control and LDR samples. Both LDR and HDR samples were significantly different from control on day 2 for early apoptotic cells, though the proportion of early apoptotic cells was relatively consistent over the length of the experiment. The proportion of cells in the mid-apoptotic stage steadily dropped, but there were no differences between sample types. HDR samples showed significant differences from both LDR and control samples 12 hours and 2 days after irradiation. Results of this assay displayed a high number of apoptotic cells in all sample types for the entire duration, with healthy, early apoptotic, and mid-apoptotic each having approximately one third of the cells. Marked cell death was not observed in the cultures using other measures of cell viability, including standard trypan blue and microscopy observations. In addition, there was no difference in cell viability between the control cells that traveled to the radiation facility and those that stayed in standard incubator conditions. It should be noted that cells measured as early and mid-apoptotic conditions with the apoptosis cell assay would appear to be viable cells through standard viability measures.

### Specific gene expression by PCR

**Fig 5:**
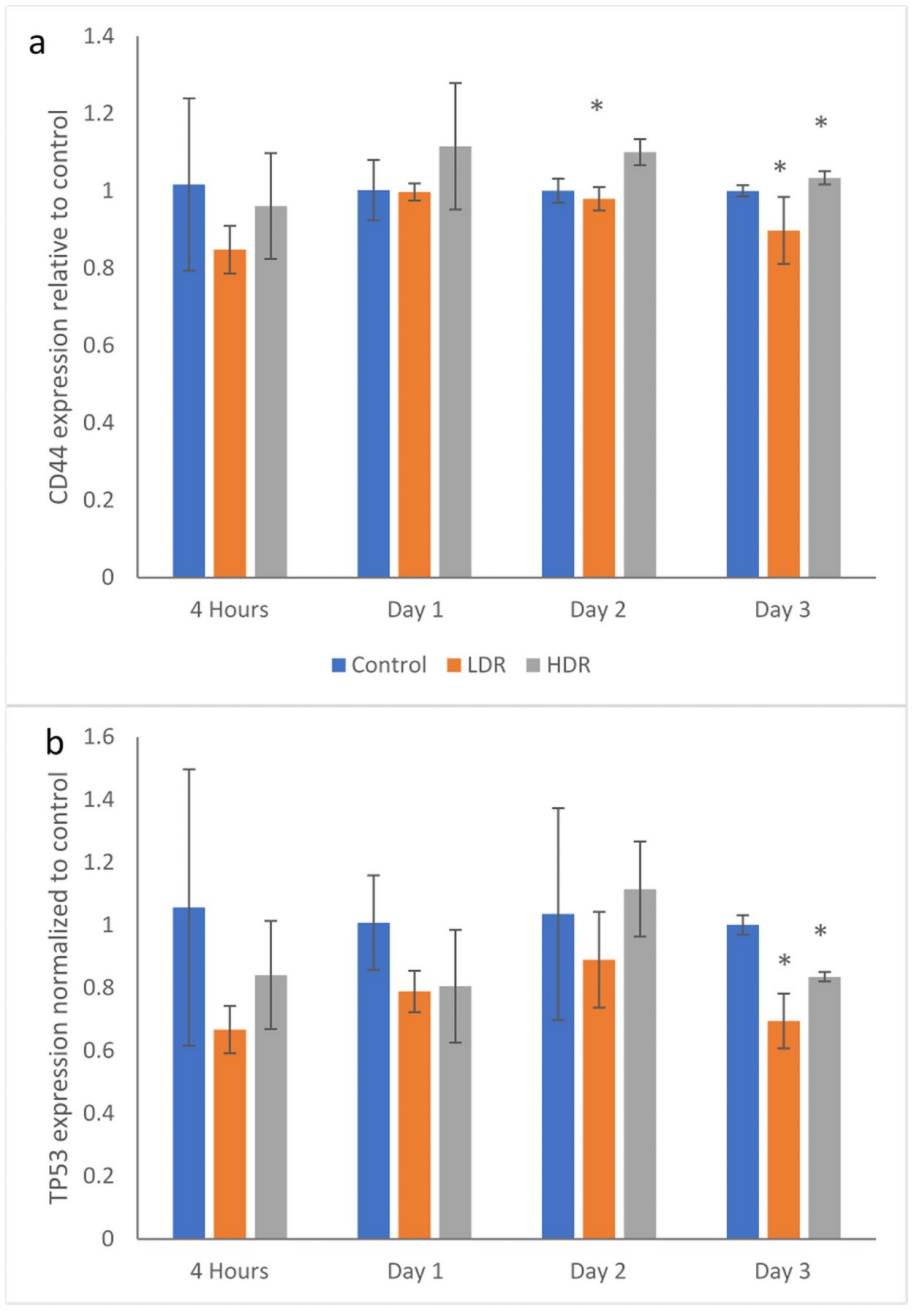
Gene expression for a) CD44 and b) TP53 in 2Gy irradiated ADSCs relative to control condition at each time point. N = 3 independent experiments for each time point and condition and * = p <0.05 relative to control.

Following irradiation, gene markers for CD44 and TP53 were checked for changes in expression using PCR. CD44 is a surface protein marker that is commonly used to characterize stem cells. Significant changes in CD44 expression could indicate the cell is losing its stemness. Fig 5a shows no significant differences between samples until day 2 when HDR has higher expression than control and LDR. On day 3, LDR and HDR are significantly different from each other, but are not different from control. TP53, also known as p53, is an important protein that is activated in response to DNA damage from various sources. In this experiment, p53 expression in irradiated samples is relatively suppressed compared to control. However, the differences are only statistically significant at day 3 where LDR and HDR are also statistically different from each other (Fig 5b).

### Immunofluorescence

**Fig 6:**
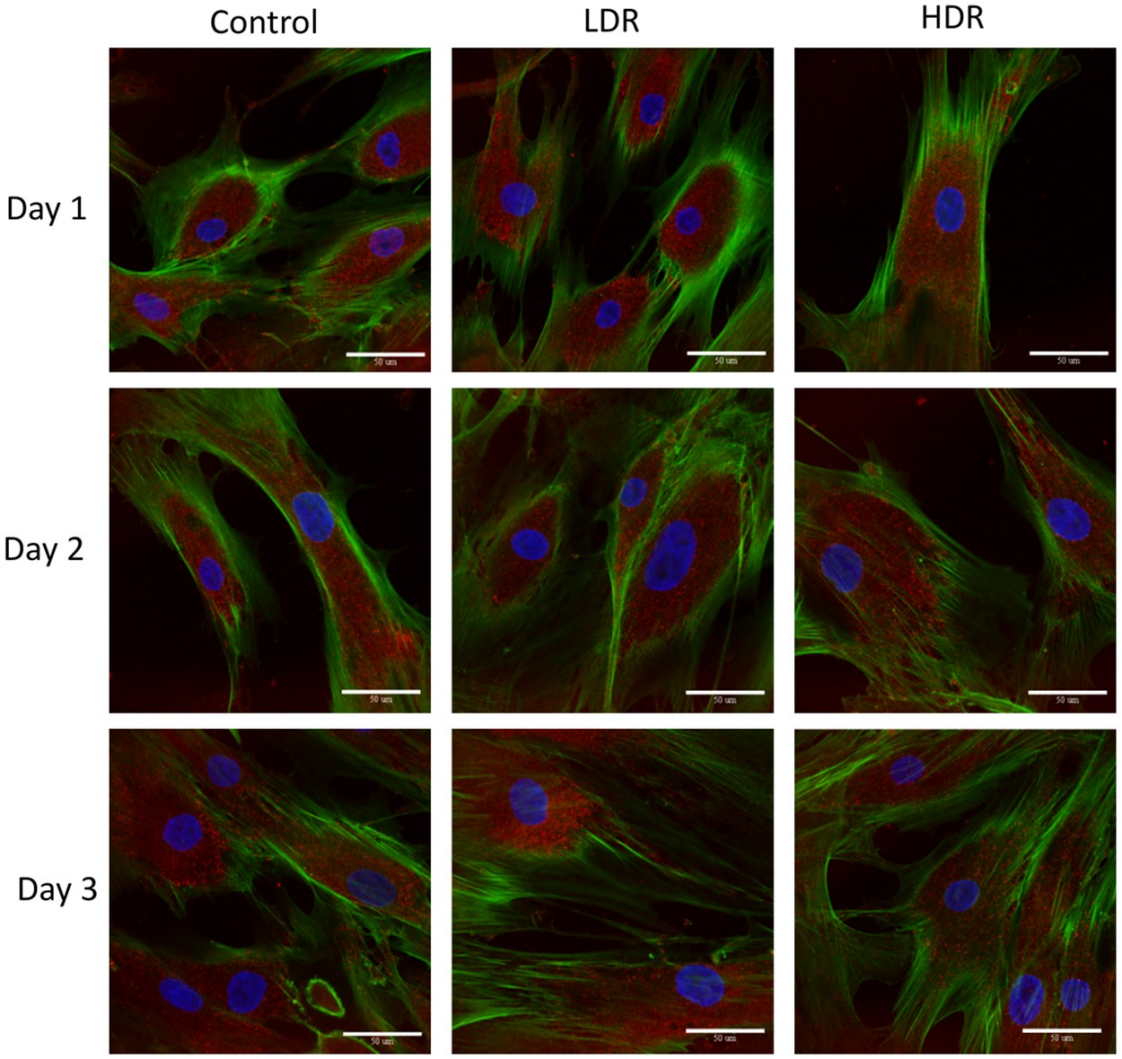
Representative images of p53 expression (red) in irradiated hADSCs, counterstained with phalloidin (green) and DAPI (blue). Images taken at 40x magnification. Bar = 50μm.

**Fig 7:**
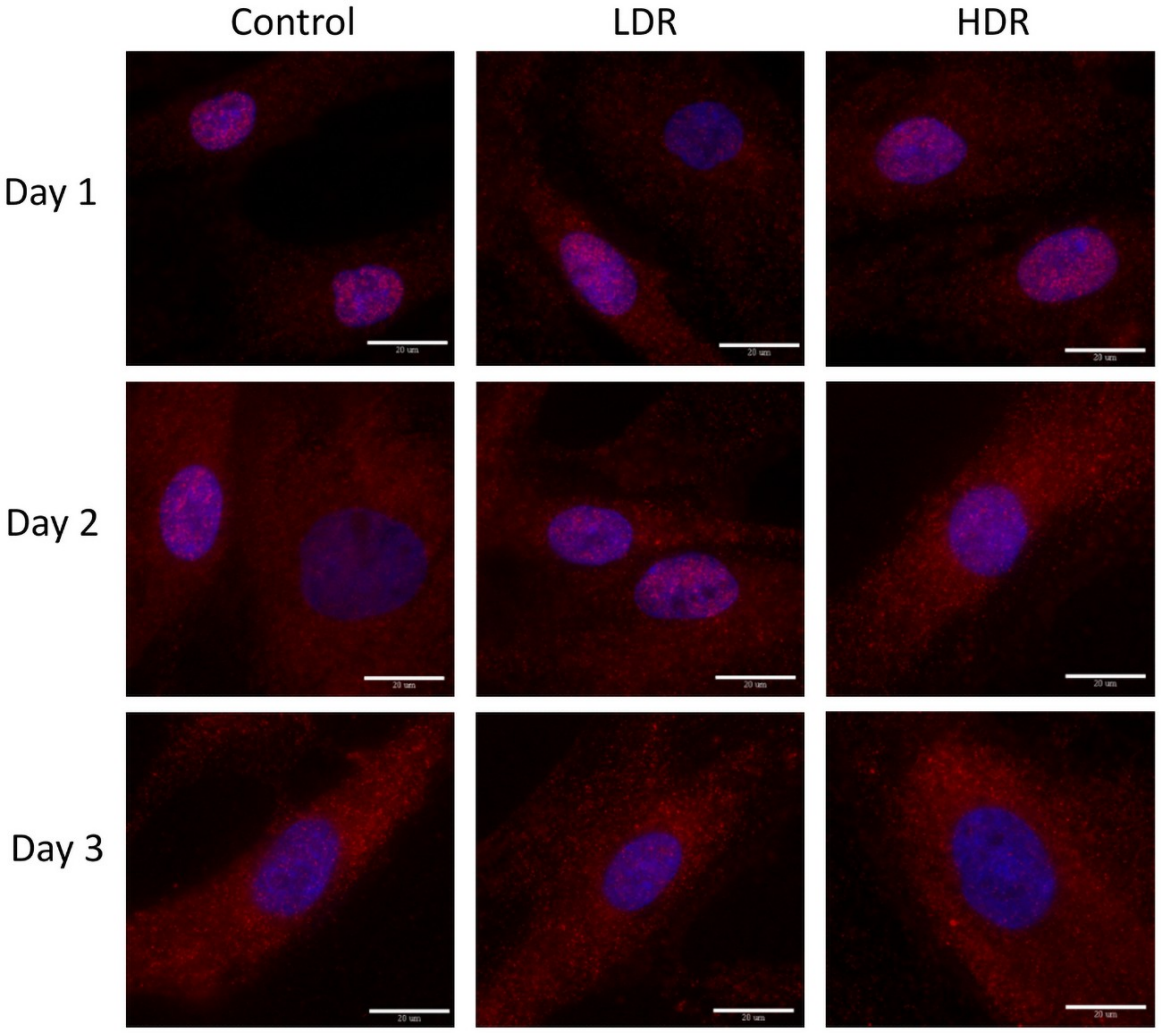
Representative images of p21 expression (red) in gamma irradiated hADSCs, counterstained with DAPI (blue). Images taken at 100x magnification. Bar = 20μm.

**Fig 8:**
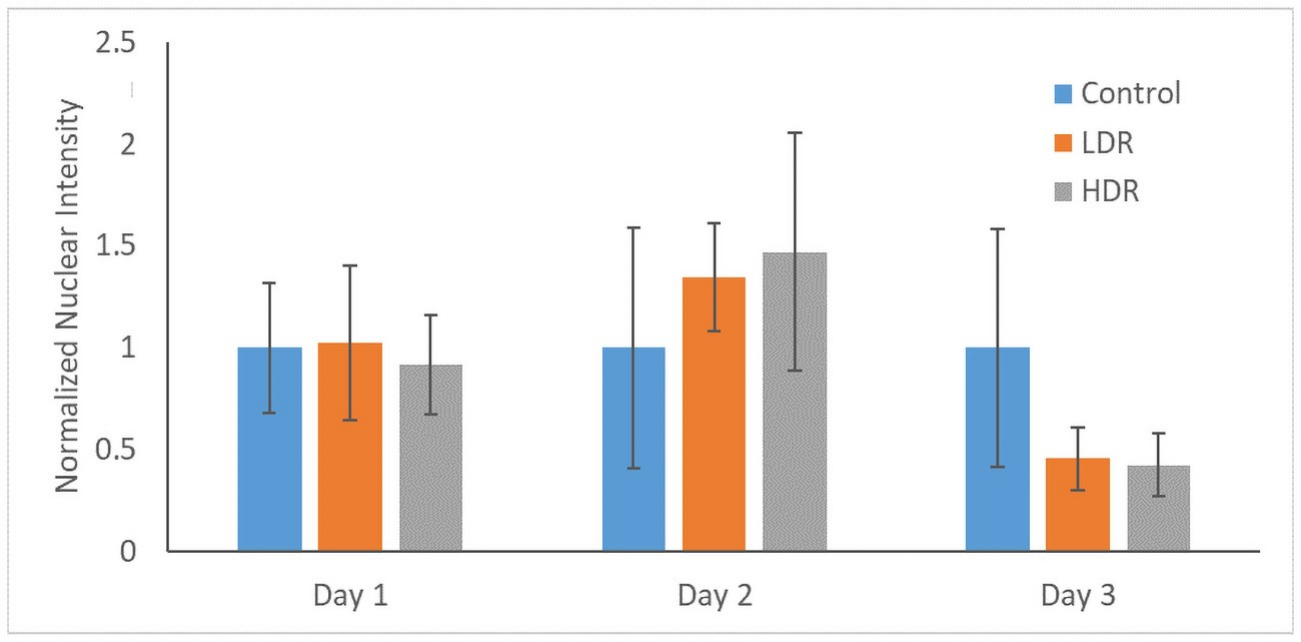
Normalized nuclear intensity of p21 expression. Data is normalized to control of each time point.

P53 and p21 are both present in the cytoplasm of all samples. Neither of these proteins are considered activated unless they are in the nucleus, otherwise they are in an inactivated, phosphorylated state. At all the time points, nuclei were found to be clear of p53 in all samples except for a few cells in day 1 controls (Fig 6). P21 shown declining activation from day 1 to day 3 in all samples. Nuclear expression is clear at day 1 and 2, but by day 3 it is difficult to distinguish between the background, cytoplasmic expression and the nuclear expression (Fig 7). To better assess differences, nuclear p21 expression was quantified from the images; intensity of the nuclear staining was normalized to the cytoplasm (N = 5–6 for each time point). The intensity of nuclear staining (Fig 8) showed no differences between samples.

### Senescence

**Fig 9:**
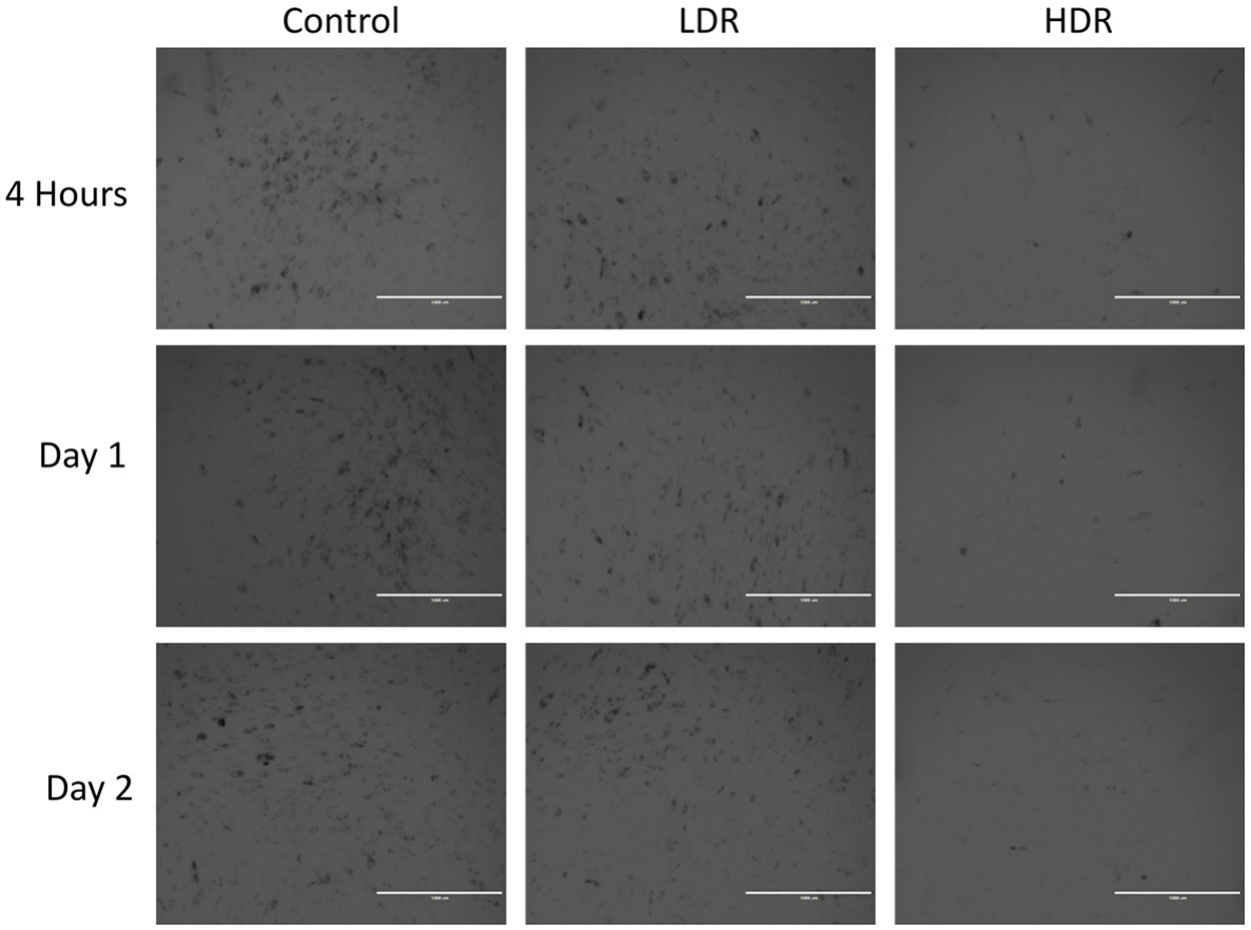
Representative imaging showing expression of β-gal representing senescent cells. Images taken at 4x magnification. Bar = 1,000μm.

Increased levels of β-gal activity, specifically senescence-associated β-gal (SA-β-gal), is a marker of senescent cells. Fig 9 shows representative images of β-gal staining in hADSCs after radiation exposure. Dark spots are high concentrations of β-gal. Control and LDR samples display high total numbers of senescent cells. HDR samples show almost no senescent cells; however, there are also fewer cells visible in culture compared to control and LDR. The numbers of β-gal dark stained cells and unstained cells were counted in each condition. The percentage of senescent cells in the images varied from 26–34% and there were no statistical differences in the percentage of senescent cells between each condition or time.

## Discussion

The purpose behind this experiment was to determine how various dose rates from a Cs^137^ gamma radiation source affect the growth and damage response mechanisms in human adipose derived stem cells. There is currently a gap in literature surrounding these cells and how they respond to radiation exposure. Considering their potential uses in stem cell therapies, the ease with which they can be acquired, because the tissue they are derived from is present all around the body, hADSCs need to be studied more in-depth [20–23]. When dealing with cancers that typically have high amounts of adipose tissue in the area, such as breast cancer, hADSCs is one of the cell types that is responsible for the recruitment of immune cells, the expression of cytokines and other molecules to help promote repair, and potential differentiation to replenish a population of depleted cells.

Following gamma radiation exposure, HDR samples experience a decrease in the number of healthy cells and the number of cells present in G_0_/G_1_ and S phase. There is also an increase in the number of cells in G_2_/M phase, which is similar to the response seen in embryonic stem cells [24,25], although the magnitude is not quite as great. LDR samples experienced a similar increase in the G_2_/M phase population, though not by the same magnitude as HDR. This response is opposite that of bone-marrow derived stem cells (BMSCs), which are another adult mesenchymal stem cells frequently used for stem cell therapy. *Chen et al* found that hBMSCs had an increase in the number of G_0_/G_1_ cells, indicating G_1_ arrest [3]. Despite hADSCs also being an adult mesenchymal stem cell line, their behavior in regard to cell cycle arrest mirrors that of embryonic stem cells much closer than other adult stem cell lines.

Cell cycle arrest after ionizing radiation is induced via the p53 pathway which is activated by the ATM kinase. Upregulation of p53 will activate p21, a cell cycle inhibitory protein, or will induce apoptosis if DNA damage is severe enough [26]. In this study, no significant upregulation of p53 was observed, either through PCR or immunocytochemistry analysis. By the later time points of the study, a significant downregulation of p53 was observed in both LDR and HDR, which has not been reported in other literature. Most studies have reported significant upregulation of p53 in the first couple hours after radiation exposure, followed by a decrease in expression back to control levels by 24 hours [24,27]. Due to the model used in the study (distance between irradiation facilities and cell analysis labs), capturing data points earlier than 4 hours after exposure was not possible. The low levels of p53 expressed in hADSCs could indicate very rapid activation then inactivation of p53 resulting in rapid activation of downstream targets relative to other cell types. Indeed, p21 activation is seen 24 hours after radiation exposure, maintaining increased expression through day 2 in both sets of irradiated samples, though no difference is seen. This expression is consistent with the continued G_2_ checkpoint arrest seen at day 3. The short term cell cycle arrest seen here is characteristic of p21; however long term exposure or high levels of p21 can also cause cellular senescence [28]. Radiation induced senescence is typically a hallmark of tumor cells, although there is no reason why healthy cells couldn’t also enter a senescent state if exposed to the right set of conditions. LDR samples in the study showed a significant number of senescent cells compared to HDR samples, suggesting that the lower dose rate may help lead to senescence induction instead of apoptosis. However, control samples also showed a high number of senescent cells, so it is more likely that the response in LDR samples is nothing more than a normal response for hADSCs.

Similar to what is seen in embryonic stem cell culture after radiation exposure [29], hADSCs in culture are never 100% arrested. Proliferation still occurs in the cells that have not been arrested due to p53/p21 activation, although a slight decrease in proliferation rate can been seen at day 3. This could be due to the significantly increased number of cells present at the G_2_ checkpoint combined with fewer apoptotic cells. The rate of proliferation is also not different from control, so the drop could just be part of the natural growth of hADSCs.

The number of differences between LDR and HDR samples in this study is minimal, although the differences seen in p53 expression and cell cycle arrest are certainly significant as these are key regulators for the recovery of the cell after radiation exposure. While the differences in the dose rates used were not as great as other studies, the rates used here are both relevant for some cancer therapies. The differences between radiation sources used for cancer treatment can vary enough that there could be an effect on the healthy cells in the tissue surrounding the tumor. The results shown here would suggest that differences in dose rate in the Gy/min range typically seen with different preclinical irradiators have a minimal effect on the function of hADSCs.
